# Rational Design of a Gd(III)–Cu(II) Nanobooster for Chemodynamic Therapy Against Cancer Cells

**DOI:** 10.3389/fchem.2022.856495

**Published:** 2022-04-07

**Authors:** Xin-Ya Shi, Ting-Xiao Shen, Ao-Lin Zhang, Li-Tao Tan, Wen-Chang Shen, Hai-Jiang Zhong, Shun-Lin Zhang, Yu-Lan Gu, Lei Shen

**Affiliations:** ^1^ Department of Oncology, Changshu No.2 People’s Hospital, Changshu, China; ^2^ Jiangsu Laboratory of Advanced Functional Materials, College of Material Engineering, Changshu Institute of Technology, Changshu, China; ^3^ State Key Laboratory of Materials-Oriented Chemical Engineering, College of Chemical Engineering, Nanjing Tech University, Nanjing, China

**Keywords:** tetrazole, Gd(III)–Cu(II), crystal structure, chemodynamic therapy, nanobooster

## Abstract

Copper (II) containing coordination complexes have attracted much attention for chemodynamic therapy (CDT) against cancer cells. In this study, the bimetallic nanobooster [Gd_2_Cu(L)_2_(H_2_O)_10_]·6H_2_O was prepared by a solvothermal method based on tetrazole carboxylic acid ligand H_4_L [H_4_L = 3,3-di (1H-tetrazol-5-yl) pentanedioic acid]. It showed considerable cytotoxicity toward three kinds of human cancer cells (HeLa, HepG2, and HT29). The MTT assay showed that the IC_50_ (half-maximal inhibitory concentration) of the complex NPs on HeLa cells (4.9 μg/ml) is superior to that of HepG2 (11.1 μg/ml) and HT29 (5.5 μg/ml). This result showed that [Gd_2_Cu(L)_2_(H_2_O)_10_]·6H_2_O NPs can inhibit cell proliferation *in vitro* and may be potential candidates for chemodynamic therapy. In addition, the cytotoxicity was also confirmed by the trypan blue staining experiment. The results promise the great potential of Gd(III)–Cu(II) for CDT against cancer cells.

## 1 Introduction

Currently, phototherapies, including photodynamic therapy (PDT), photothermal therapy (PTT), and chemodynamic therapy (CDT) have received tremendous attention as advanced methods for cancer treatment. Both the treatments can induce cancer cell apoptosis by producing reactive oxygen species (ROS) (Palao et al., 2016; Lin et al., 2018; Guo et al., 2020; Jia et al., 2022). Among these, phototherapy is restricted to further application in clinical treatment due to the limitation of tissue light penetration and hypoxic environment. CDT does not rely on light and only uses copper or iron to catalyze endogenous H_2_O_2_ *via* a Fenton-like reaction to produce hydroxyl radicals (•OH) to kill cancer cells. For example, Cao et al. designed a Mn–Cu bimetallic complex for CDT; this complex can efficiently generate ROS and reduce the glutathione (GSH) level so as to improve the CDT effect (Cao et al., 2019).

Coordination complexes have been designed and developed as multifunctional materials for cancer treatment (Yang et al., 2014; Yu et al., 2021). The tetrazole carboxylic acid ligand has two functional groups: tetrazole ring and carboxyl group, which enable it to have excellent coordination ability and more coordination modes with metal ions. 1) Ligands containing nitrogen and oxygen atoms provide the possibility of regulating the final supramolecular structure due to the diversity of coordination modes with metals. 2) The diverse coordination modes of tetrazole and carboxylate groups can form various different coordination connection modes. 3) Abundant N and O atoms can participate in the formation of hydrogen bonds, which can stabilize the supramolecular assembly. Multiple modes of monodentate coordination, chelate coordination, and bridged coordination can be realized. Therefore, the research on tetrazole carboxylic acid complexes is of great significance (Aromí et al., 2011; Fan et al., 2021; Liu et al., 2012; Sun et al., 2012; Huang et al., 2013; Li et al., 2013; Chen et al., 2014; Liu et al., 2015; Liu et al., 2016; Wu et al., 2011). At present, the synthesis and structure of a variety of tetrazole carboxylic acids and their coordination complexes and properties have been studied, showing their important application value in the fields of molecular magnetism, molecular absorption, and catalysis (Wriedt et al., 2012; Zou et al., 2014; Wu et al., 2015; Yang et al., 2015; Zou et al., 2015). With the in-depth study of tetrazole carboxylic acid complexes, it is found that they have special biological activities against human tumor cells (Wriedt et al., 2013; Li et al., 2015; Sun et al., 2016; Zhu et al., 2016).

**SCHEME 1:**
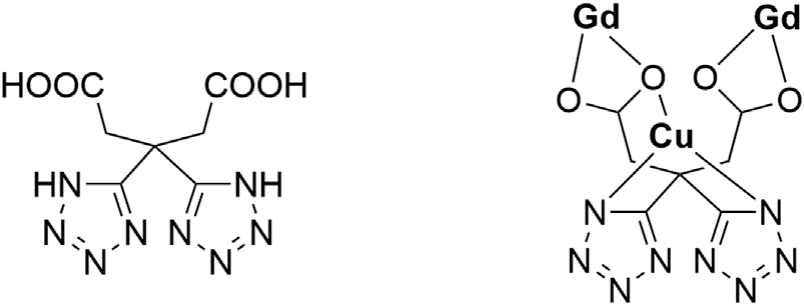
Drawing for H_4_L ligand.

In previous studies, we have reported a series of tetrazole carboxylate–based complexes as nanoboosters, which can boost O_2_ or H_2_O_2_ to generate ROS to induce cancer cell apoptosis, exhibiting high toxicity and excellent biocompatibility for photodynamic therapy (Yang G et al., 2018; Zhai et al., 2018; Yang et al., 2019; Zhu et al., 2019; Xu et al., 2020; Yang et al., 2020). As an extension of our research, the tetrazole carboxylic acid ligand–based H_4_L (Scheme 1) was selected for self-assembly with Gd(III)–Cu(II) ions, and a new heteronuclear complex [Gd_2_Cu(L)_2_(H_2_O)_10_]·6H_2_O was obtained with good antitumor properties (Scheme 1). The complex structure was characterized by X-ray single crystal diffraction, and its structure and properties were analyzed to find its potential application. The NPs of the complex were prepared by a nanocoprecipitation method with PEG-_2000_ (polyethylene glycol) to behave as a booster that is capable of boosting H_2_O_2_ to produce •OH to induce apoptosis. Human cervical cancer cells (HeLa), human hepatoma cells (HepG2), and human colorectal adenocarcinoma cells (HT29) were selected to investigate the chemodynamic therapy efficacy *in vitro*. In addition, the half-maximal inhibitory concentration (IC_50_) of 4.9 μg/ml, 11.1 μg/ml, and 5.5 μg/ml with irradiation was observed in the MTT assay. The results showed that [Gd_2_Cu(L)_2_(H_2_O)_10_]·6H_2_O NPs can inhibit cell proliferation in three kinds of tumor cells, and have the lowest IC_50_ value for HeLa cells, which may be potential candidates for chemodynamic therapy.

## 2 Experimental Section

### 2.1 Materials and Methods

The H_4_L ligand was prepared according to the literature methods (Lin et al., 2008). All commercially available chemicals of analytical grade were used directly. The FT-IR spectra as the KBr disk were recorded on a Nicolet-IS10 spectrometer. The elemental analysis of CHNO was conducted by using a EA1110 CHNO-S micro analyzer. Luminescence properties were analyzed by using a Hitachi F-7000 fluorescence spectrophotometer. Powder X-ray diffraction (PXRD) measurements were carried out by using a Rigaku D/MAX 2200 diffractometer. Scanning electron microscopy (SEM) was performed using a Hitachi S-4800. UV–Vis spectroscopy was conducted using a Shimadzu UV-3600 spectrophotometer. Single crystal X-ray diffraction was carried out using a Bruker SMART APEX II DUO diffractometer. The cellular images were recorded by using a Bruker *In Vivo* Imaging System Fx Pro.

### 2.2 Synthesis of [Gd_2_Cu(L)_2_(H_2_O)_10_]·6H_2_O

0.1 mmol (0.0268 g) H_4_L was dissolved in 6 ml distilled water. The solution was adjusted to pH = 6 using 2% KOH. Then, 0.4 mmol (0.0451 g) Gd(NO_3_)_3_·6H_2_O, 0.1 mmol (0.0242 g) Cu(NO_3_)_2_·3H_2_O, and 1 ml of EtOH were added to it. Then, the solution was heated for 48 h at 130 °C in a stainless steel reactor lined with Teflon. After cooling to room temperature, blue block-shape crystals of [Gd_2_Cu(L)_2_(H_2_O)_10_]·6H_2_O were obtained by filtration and washed with EtOH. Yield: 59% based on Cu^2+^. Anal. calculated for C_14_H_40_CuGd_2_N_16_O_24_: C 14.08; H 3.38; N 18.76% found: C 14.32; H 3.36; N 18.48%. IR (KBr, cm^−1^) 3412.95 (s), 2367.31 (w), 1566.55 (m), 1411.36 (m), 1295.05 (s), 1237.85 (w), 1142.94 (w), 1027.44 (w), 922.03 (w), 660.89 (w), 624.05 (w).

### 2.3 X-Ray Crystallography

The structure of the complex synthesized in the experiment was studied by using X-ray single-crystal diffraction. Crystallographic data for the complex were recorded on a Bruker Apex II DUO diffractometer with a Mo–*K*α source (*λ* = 0.71073 Å) at room temperature. The relevant data were collected and full matrix least squares correction was performed with the direct method (SHELXTL-2014) to analyze the crystal structure (Sheldrick, 2008). Table 1 shows the crystallization parameter data of the target complex, and main bond lengths and angles and relevant hydrogen bond parameter data are shown in Table S1 and Table S2 (ESI†).

**TABLE 1:** Crystallographic parameters of [Gd_2_Cu(L)_2_H_2_O_10_]·6H_2_O.

Empirical formula	C_14_H_28_CuGd_2_N_16_O_18_·6H_2_O
Formula mass	1194.66
Crystal system	Monoclinic
Space group	P21/n
*a* (Å)	10.4322 (4)
*b* (Å)	9.8578 (4)
*c* (Å)	19.1734 (7)
*α* (°)	90.00
*β* (°)	104.0440 (4)
*γ* (°)	90.00
*V* (Å^3^)	1912.83 (13)
*Z*	2
*T*/K	296
D_calcd (_g.cm^−3^)	2.074
*μ* (mm^−1^)	4.084
Reflections collected	19693
Unique reflections (R_int_)	3878 (0.0192)
No. of observations (I > 2.00)	3680
No. of variables	259
R_1_ ^a^ wR_2_ ^b^ (I > 2sigma(I))	0.0177, 0.0450
R_1_, wR_2_ (all data)	0.0189, 0.0458
GOF^c^	0.990
Δ/ρ_max_ (e/Å^3^)	0.537
Δ/ρ_min_ (e/Å^3^)	−0.631

a R = Σ||F_o_|-|F_c_|/Σ |F_o_|.

b Rw = {Σ w (F_o_^2^-F_c_^2^)^2^/Σ w (F_o_^2^)^2^}^1/2^.

c GOF = {w (F_o_^2^-F_c_^2^)^2^)/(n-p)}^1/2^, where n = number of reflections and p = total numbers of parameters refined.

### 2.4 Preparation of the Complex Nanoparticles

Due to the poor solubility of the complex, the NPs with good dispersibility in water were prepared by nanocoprecipitation with PEG_-2000_ by a “bottom–up” method, and this can be universally found in the literature (Li et al., 2017; Yang J et al., 2018). For a typical experiment, tetrahydrofuran (THF, 1 ml) solution containing the complex (2 mg) and PEG_-2000_ (5 mg) was sonicated into distilled water. After stirring for 10 min, N_2_ was bubbled for 20 min to remove THF. The solution was then stored in the dark for further use (Yang J et al., 2018). Finally, NPs of the complex in the solution were obtained by centrifugation. The as-prepared NPs have good dispersibility in water. In addition, nanoparticles have enhanced permeability and retention (EPR) effect and can passively target the tumor to enhance uptake.

### 2.5 Cell Culture and MTT Assay

HeLa, HepG2, and HT29 cell lines were obtained from the Cell Bank of SIBCB, CAS (China). These cells were cultured in the minimum essential medium (DMEM, Gibco; Thermo Fisher Scientific) with 10% fetal bovine serum (FBS; Gibco; Thermo Fisher Scientific). All the culture media contain 100 units/mL penicillin and 100 μg/ml streptomycin. The cells were cultured at 37°C in a humidified incubator with 5% CO_2_.

HeLa, HepG2, and HT29 cell lines were seeded in 96-well plates at a density of 1 × 10^5^ cells/ml, 5 × 10^4^ cells/ml, and 5 × 10^4^ cells/ml, respectively, for 12 h to get attached. Cell viability assays of the NPs were conducted by first dissolving in distilled water, which were diluted with DMEM to various concentrations and transferred in the 96-well plate with the same volume (200 µL) in each well for 24 h. Then, the plate was irradiated with a xenon lamp (30 mW/cm^2^) for 5 min. Cell viability was determined by the MTT [3-(4,5-dimethylthiazol-2-yl)-2,5-diphenyltetrazolium bromide] assay. MTT in PBS (5 mg/ml, 20 μL) was added to each well and incubated for 3 h under the same conditions at 37°C. Then, the medium was removed and 200 ml DMSO was added. The plate was agitated on a Bio-Tek microplate reader at ambient temperature. The average absorbance of the no cell line was subtracted from the readings of the other wells. The cell viability was calculated by the following equation: cell viability (%) = mean absorbance in each group incubated with different concentrations of NPs/mean absorbance in the control group. The average absorbance of the blank well (no cells) was removed from the data of the other wells.

### 2.6 Measurement of •OH Generation

To investigate the •OH generation, the solution of the complex NPs (0.5 ml, 10 μg/ml) was added into methylene blue (MB, a dye that can be faded by •OH) solution (0.5 ml, 20 μg/ml) to obtain a mixed solution and allowed to stand at room temperature for 30 min. The absorbance value of the MB and mixed solution of MB with NPs was recorded at about 665 nm *via* UV–Vis spectroscopy.

## 3 Results and Discussions

### 3.1 Synthesis and Characterization of Complex NPs

In weak acidic conditions, hydrothermal reactions of H_4_L and metal salt with a ratio of 1:5 in MeOH-H_2_O at 130°C for 2 days provided the complex in moderate of 59%. Crystals of the complex are all air-stable. The elemental analysis reveals that the component is in well-agreement with the results of the X-ray diffraction analysis.

The main IR peaks of the complex are as follows: the absorption peak at 3412.9 cm^−1^ is attributed to water molecules, the peak at 2367.3 cm^−1^ belongs to the carboxyl peak, and peaks within 1566.5 cm^−1^ to 1411.3 cm^−1^ are attributed to the tetrazole ring conjugate system of the ligand. The results of the infrared analysis are in well-accordance with the composition of the complex.

**FIGURE 1:**
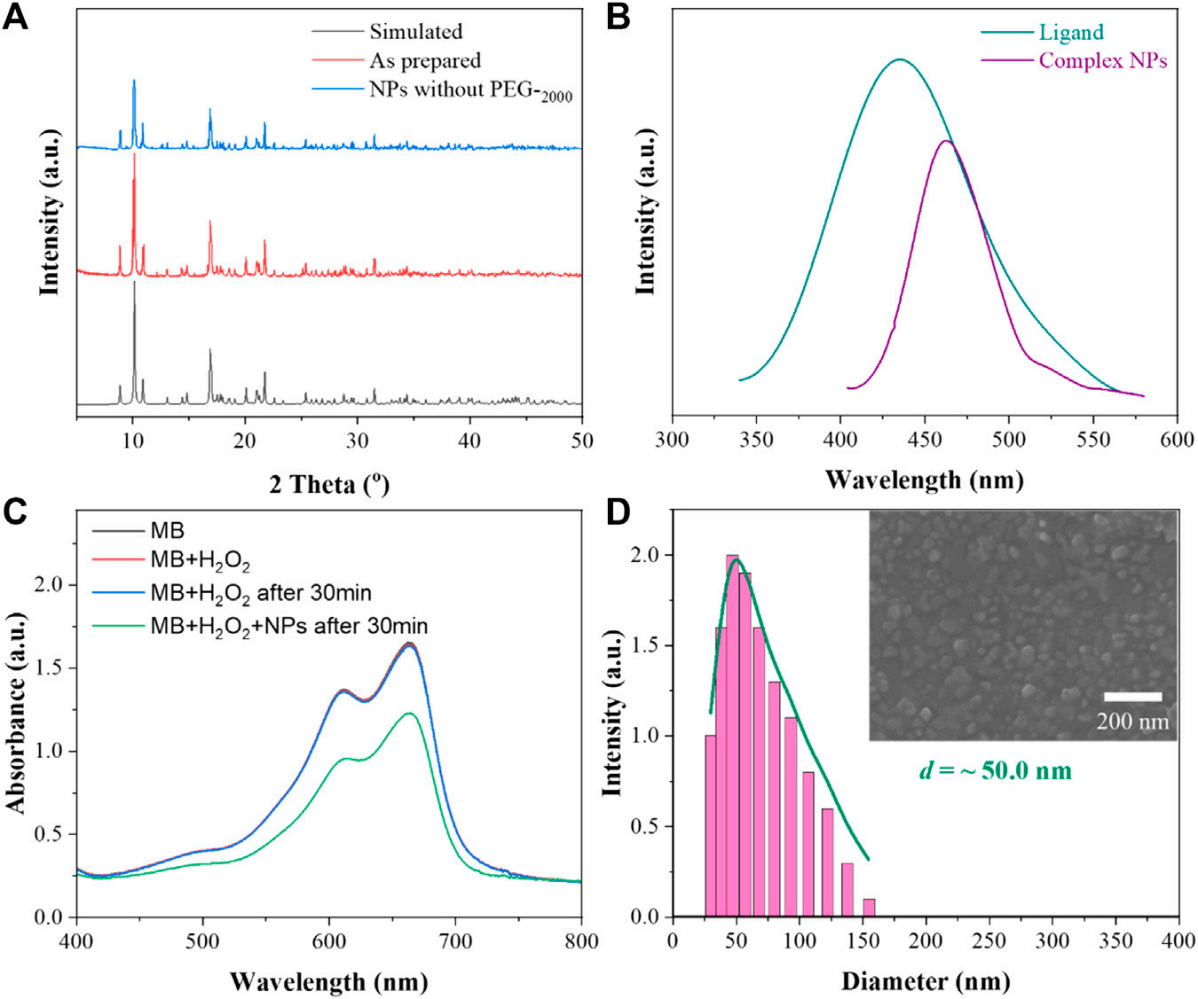
**(A)** XRD patterns of the complex; **(B)** schematic diagram of the fluorescence emission spectrum of the ligand and complex NPs; **(C)** MB degradation in the presence of H_2_O_2_ with the complex NPs for the detection of generation of hydroxyl; **(D)** DLS and SEM images of the complex NPs.

X-ray diffraction (XRD) was used to characterize the stability of the complex NPs in water. As shown in Figure 1A, the patterns are exactly identical to those simulated from single-crystal analysis, indicating the high phase purity of the bulk crystal. Notably, the PEG-free complex NPs were prepared by the same method, maintaining the structural integrity of the framework after exposure to water at 25°C for 1 day and demonstrating excellent stability of these complex NPs.

The fluorescence properties of the ligand H_4_L and complex NPs were studied by using a fluorescence spectrophotometer at room temperature. As shown in Figure 1B, through the exploration of its emission peak, the ligand H_4_L has the largest characteristic emission peak at 426 nm with 374 nm excitation. With 311 nm excitation, the complex has a maximum emission peak at 469 nm. The observed red shift of 43 nm for the complex is in good agreement with the previously reported complex (Guo et al., 2018). The shifts are probably due to π*–π transitions of the corresponding ligand because similar peaks also appear for the free ligand.

Based on the Fenton-like reaction mechanism, Cu(II) complex NPs can catalyze H_2_O_2_ to •OH (Liu C et al., 2019; Bao et al., 2020). In order to study the catalytic performance of the complex NPs, MB was selected as an indicator of ROS in the presence of H_2_O_2_. As shown in Figure 1C, the absorbance of MB decreased *via* oxidation when the complex NPs were added, indicating the effective generation of •OH. In contrast, no obvious absorbance decrease was observed without NPs even after 30 min. The Gd(III)–Cu(II) complex can also oxidize GSH to GSSG to further enhance the yield of ROS.

Nanocoprecipitation was used to improve the aqueous solution dispersability of the NPs. SEM (scanning electron microscope) and DLS (dynamic light scattering) were used to characterize the size and diameter of the complex NPs. As shown from Figure 1D, it can be seen that the complex can self-assemble NPs with good dispersion, and the average diameter is about 50 nm, suggesting the suitability for EPR. In addition, the stability of the complex in PBS (pH = 5.5 and 7.4) and DMEM with 10% FBS was also determined. The complex NPs without PEG_-2000_ were retained in the aqueous solutions at RT for 1 day. The diffraction patterns are similar to the simulated ones (Supplementary Figure S1, ESI†), suggesting the high-phase purity of the bulk products. In addition, no obvious changes were observed after incubation with the NPs in PBS or in DMEM, indicating their good stability. Moreover, the DLS results showed the diameter of the NPs with a slight aggregation in water, PBS, and DMEM (Supplementary Figure S2, ESI†). This may be due to the low solubility of NPs in aqueous solution and absence of PEG_−2000_ coating.

### 3.2 Crystal Structure of [Gd_2_Cu(L)_2_(H_2_O)_10_]·6H_2_O

**FIGURE 2:**
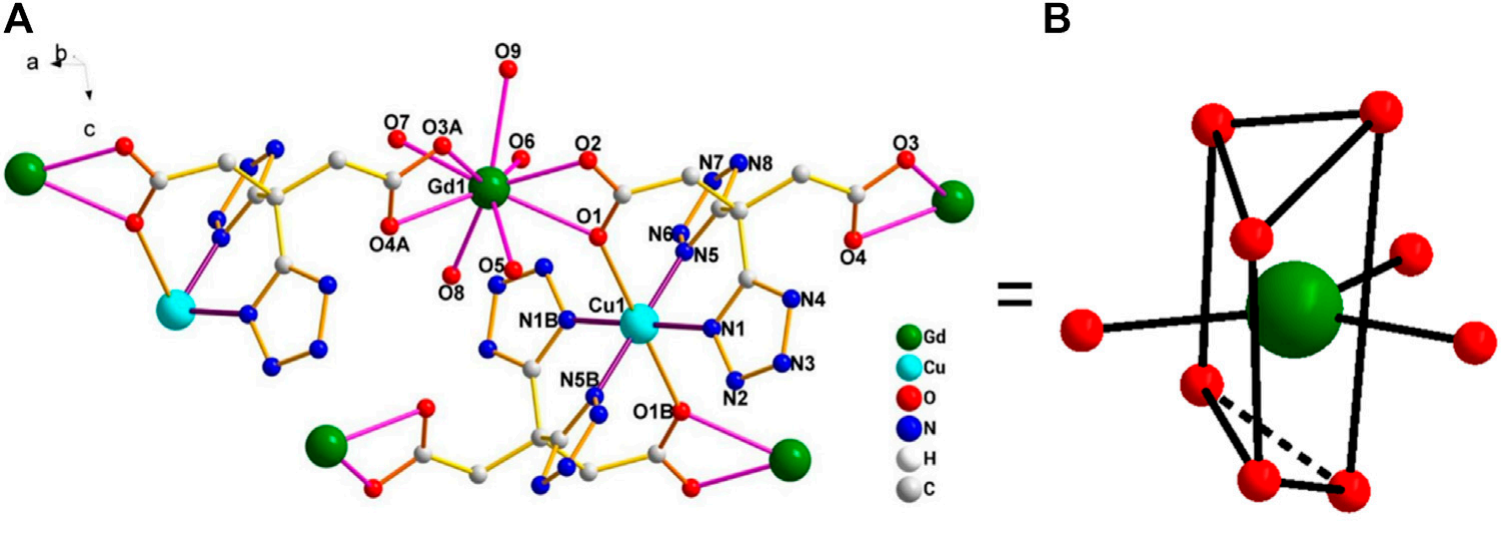
**(A)** Diagram of the coordination environment of the Gd(III) and Cu(II) center in the complex [Gd_2_Cu(L)_2_(H_2_O)_10_]·6H_2_O. **(B)** Distorted tricapped trigonal prism of Gd(III).

**FIGURE 3:**
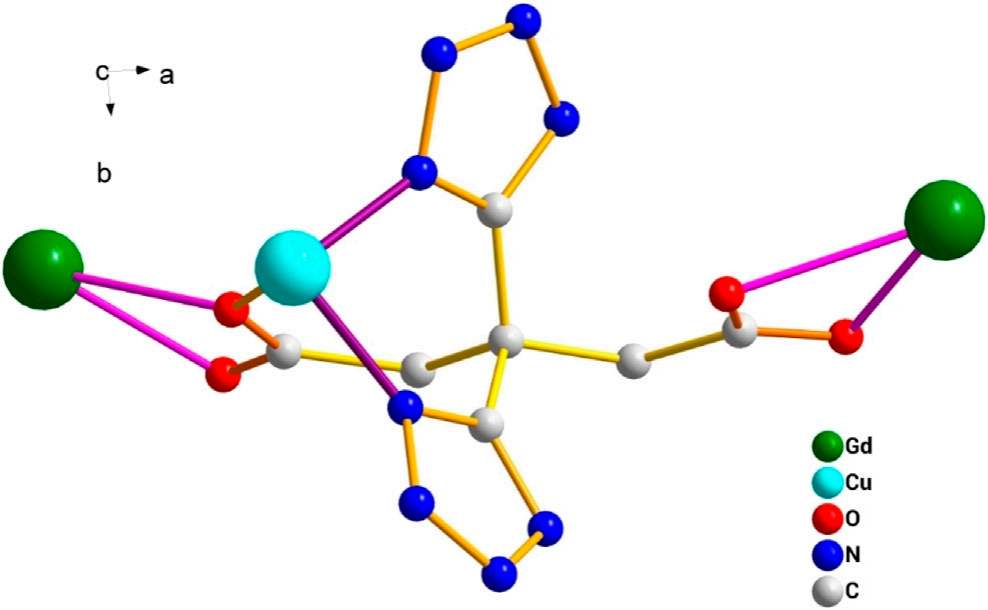
Coordination mode diagram of the H_4_L.

**FIGURE 4:**
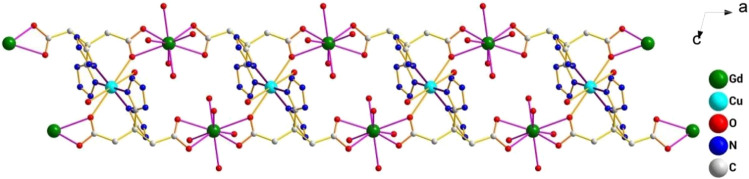
One-dimensional structure of the complex [Gd_2_Cu(L)_2_(H_2_O)_10_]·6H_2_O observed along the b-axis direction.

**FIGURE 5:**
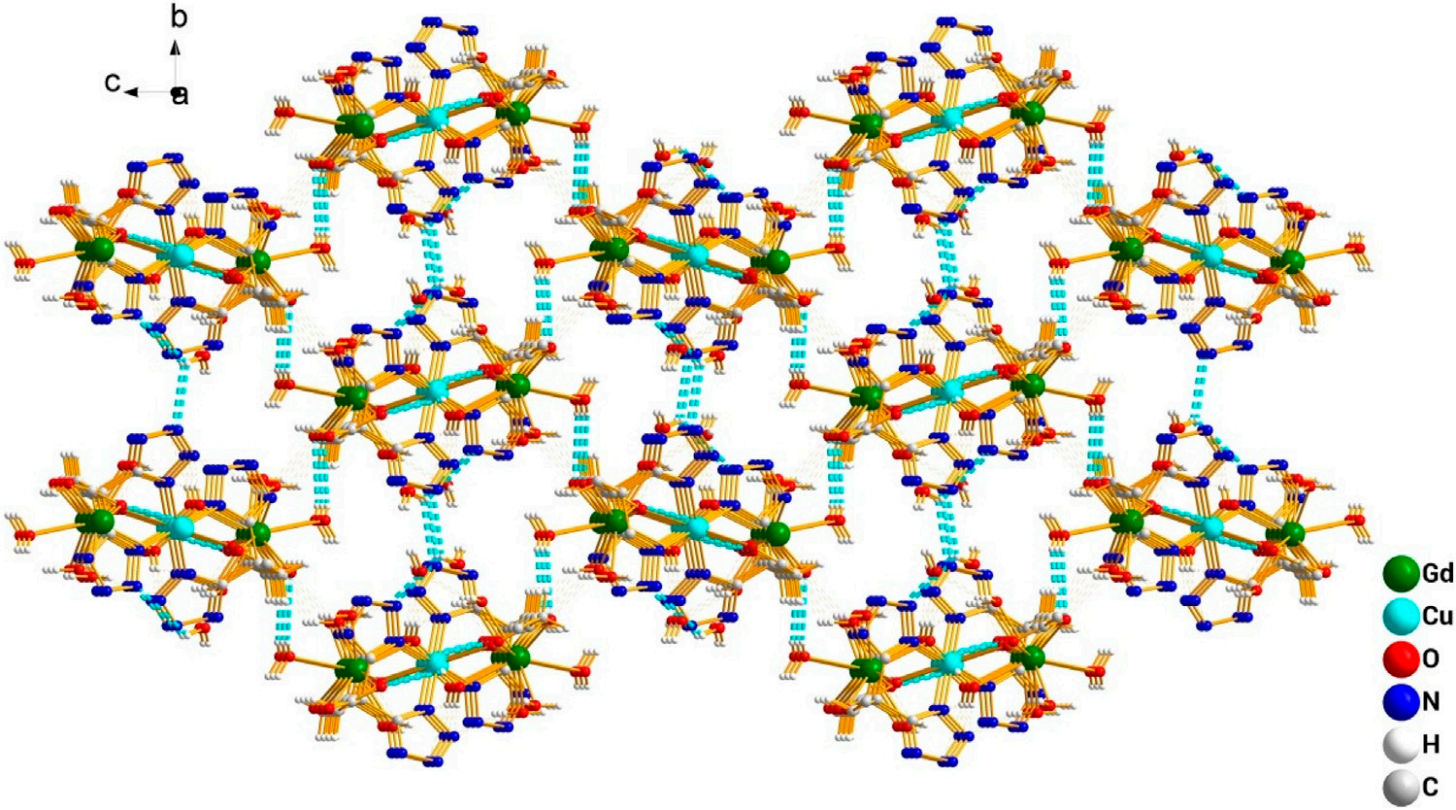
3D structure diagram of the complex [Gd_2_Cu(L)_2_(H_2_O)_10_]·6H_2_O formed by hydrogen bond interaction.

This complex belongs to the monoclinic space group *P2*_1_/n with a crystallographic ally-independent symmetric unit. From Figure 2, each Gd(III) atom is nine-coordinated by four oxygen atoms (O1, O2, O3A, and O4A) from two carboxylate groups and five oxygen atoms (O5, O6, O7, O8, and O9) from five water molecules, thus forming a deformed and distorted tricapped trigonal prism coordination configuration. The Cu(II) cation is coordinated with four nitrogen atoms (N1, N5, N1B, and N5B) of the tetrazole ring and two carboxyl oxygen atoms (O1 and O1B) from two L^4−^ ions, forming a deformed octahedron coordination configuration. In addition, four oxygen atoms from two carboxyl groups of L^4−^ chelated with two Gd(III) ions (Figure 3), and two N atoms from two tetrazole rings and a carboxyl oxygen atom connected with one Cu(II) ion (Scheme 1). By analyzing the bond lengths of the complex, the bond lengths of Gd–O are 2.391–2.544 Å and that of Cu–O are 2.492 Å. The bond lengths between the Cu atom and N atom are in the range of 1.976–1.992 Å. As shown in Figure 4, two such ligands set up a double bridge between two Cu(II) ions and two Gd(III) ions to generate a bimetallacycle of [Cu_2_Gd_2_(L)_2_]. The adjacent bimetallacycle is further connected to yield a one-dimensional chain structure, and by hydrogen bonding between the chains, a three-dimensional supramolecular structure was formed (Figure 5).

### 3.3 Cytotoxicity and Trypan Blue Staining

**FIGURE 6:**
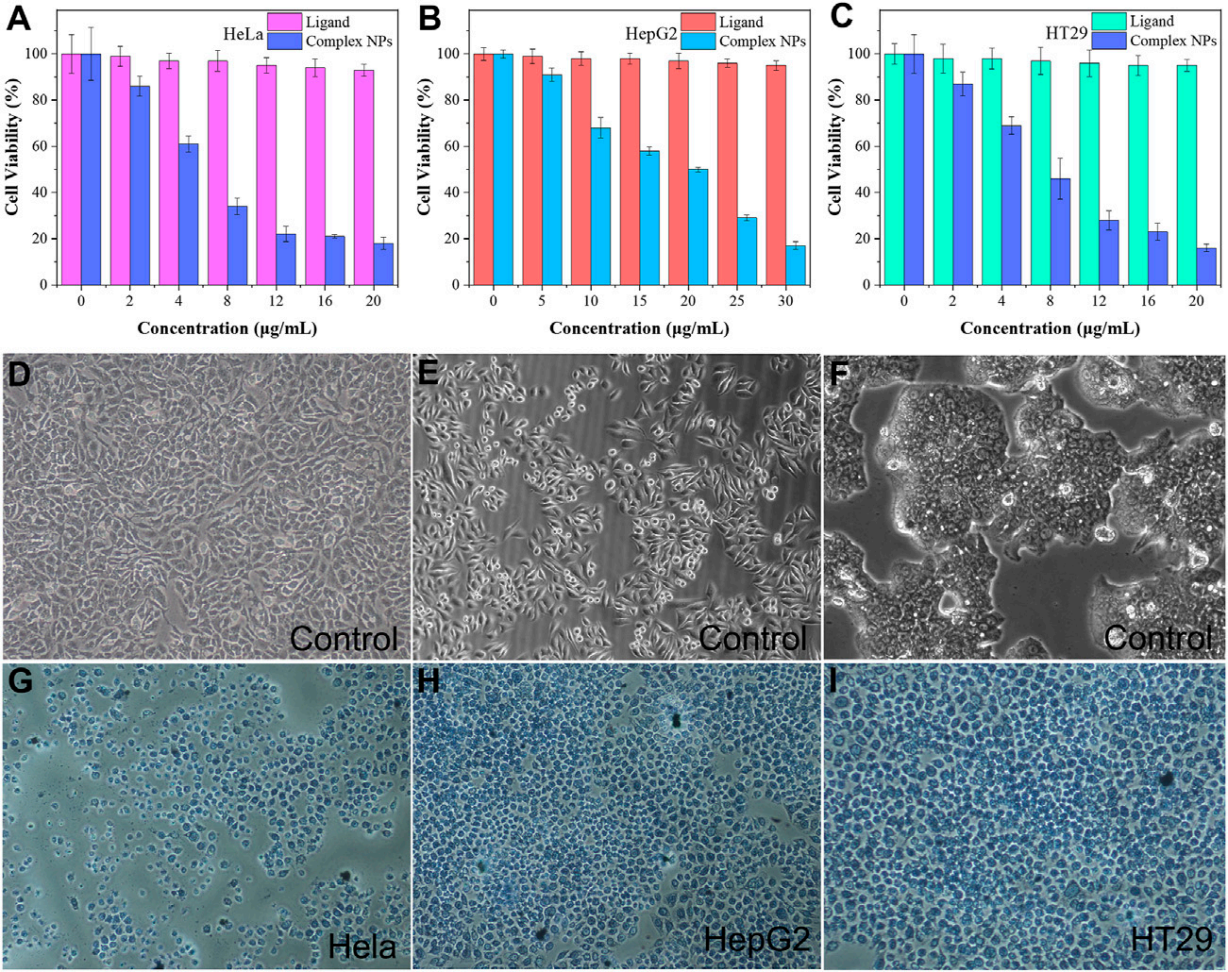
**(A–C)** In vitro MTT assay of HeLa, HepG2, and HT29 cells was treated with ligand H4L and complex NPs; trypan blue fluorescent staining of the control group for **(D)** HeLa cells, **(E)** HepG2 cells, and **(F)** HT29 cells; Complex NPs for **(G)** HeLa cells **(H)** HepG2 cells **(I)** HT29 cells.

The MTT assay was performed to further investigate the cytotoxicity of the complex NPs. Different concentrations of NPs of the complex were cultured with HeLa, HepG2, and HT29 cells. As shown in Figures 6A–C, the cell viability of the group cultured with H_4_L remained high even at high concentration, which demonstrated the low cytotoxicity of the ligand. The complex NPs displayed promising cytotoxic activity against all the three cancer cell lines. As the NPs concentration increases, the cell viability decreases, and the IC_50_ values of HeLa, HepG2, and HT29 cells are calculated to be approximately 4.9 μg/ml (0.90 μM), 11.1 μg/ml (2.05 μM), and 5.5 μg/ml (1.01 μM), respectively. Among them, the IC_50_ of the complex for HeLa was the lowest, which is superior to that of the previously reported Cu complexes with tetrazole (triazole)–carboxylate as ligands, such as [Cu(2-pytzipa)_2_(H_2_O)_2_]·2H_2_O (7 μM) (Zhai et al., 2017), [Cu(atzpa)_2_], [Cu(pytzipa)_2_], [Cu_4_(Hphtz)_8_](ClO_4_)_4_·4H_2_O (Zhang et al., 2021), [Cu(L^1^)_2_(ClO_4_)_2_]∙2MeCN [Cu(L^1^)_2_(MeOH)_2_](ClO_4_)_2_, and [Cu(L^3^)_2_(NO_3_)_2_]∙3H_2_O (Li et al., 2021) (Table 2), and also superior to various transition metal complexes based on tetrazole–carboxylate, such as [Zn (pytzipa)_2_(H_2_O)_4_]·2H_2_O (35 μg/ml) (Gu et al., 2018), Ca (2-pytzipa)_2_(H_2_O)_4_] (48 μg/ml), and [Ca(3-pytzipa)_2_(H_2_O)_2_]n (30 μg/ml) (Cao et al., 2019). This result also demonstrated that the Cu(II)-containing complex was able to effectively decrease the GSH level in the solution to induce cell apoptosis (Tang et al., 2019). On the contrary, the overexpression of GSH can reduce the reaction of Cu(II) to Cu(I) *via* Fenton-like reaction, further improving the production rate of •OH and reducing the antioxidant capacity of tumor cells (Liu Y et al., 2019). From the abovementioned fact, the Gd(III)–Cu(II) complex showed better apoptosis-inducing effect than most copper compounds; this may be because the addition of Gd(III) increased the uptake of NPs by cancer cells, resulting in a more efficient CDT therapeutic effect (Lee et al., 2015; Ren et al., 2019; Zeng et al., 2020; Stinnett et al., 2021).

**TABLE 2:** Comparison of cytotoxicity with other Cu(II) complexes for HeLa cells.

Complex	IC_50_ (μg/ml)	Reference
[Cu(atzpa)_2_]	41.45	Zhang et al. (2021)
[Cu(pytzipa)_2_]	33.76	Zhang et al. (2021)
[Cu_4_(Hphtz)_8_](ClO_4_)_4_·4H_2_O	28.92	Zhang et al. (2021)
[Cu(L^1^)_2_(ClO_4_)_2_]∙2MeCN	92.3	Li et al. (2021)
[Cu(L^1^)_2_ (MeOH)_2_](ClO_4_)_2_	67.5	Li et al. (2021)
[Cu(L^3^)_2_(NO_3_)_2_]∙3H_2_O	73.7	Li et al. (2021)
[Gd_2_Cu(L)_2_(H_2_O)_10_]·6H_2_O	4.9	This work

After trypan blue staining, the cell nucleus incubated with the NPs showed a stronger blue–white color than the control group, indicating that it had a significant apoptosis-inducing effect to HeLa, HepG2, and HT29 cells (Figures 6G–I). From the comparison of these cell morphologies, it can be seen that the morphology of live cells in control groups is irregular, while that of dead cells tends to be regular. In addition, the volume of the dead cells had generally shrunk, possibly due to the mechanism of apoptosis rather than necrosis (Cao et al., 2019).

## 4 Conclusion

In conclusion, a new nanobooster [Gd_2_Cu(L)_2_(H_2_O)_10_]·6H_2_O was designed and synthesized by the solvothermal reaction. It showed a one-dimensional chain structure and was capable of catalyzing H_2_O_2_ to form cytotoxic hydroxyl radicals, indicating its excellent cytotoxicity against the HeLa, HepG2, and HT29 cells as confirmed by the MTT assay and trypan blue staining. HeLa cells are the most sensitive to the NPs. The bimetallic complex has potential in chemodynamic therapy against human cervical, liver, and colon carcinoma cells.

## Data Availability

Publicly available datasets were analyzed in this study. These data can be found here: CCDC 2103482.
