# Corrigendum: Rational design of a Gd(III)–Cu(II) nanobooster for chemodynamic therapy against cancer cells

**DOI:** 10.3389/fchem.2022.1075376

**Published:** 2022-11-22

**Authors:** Xin-Ya Shi, Ting-Xiao Shen, Ao-Lin Zhang, Li-Tao Tan, Wen-Chang Shen, Hai-Jiang Zhong, Shun-Lin Zhang, Yu-Lan Gu, Lei Shen

**Affiliations:** ^1^ Department of Oncology, Changshu No. 2 People’s Hospital, Changshu, China; ^2^ Jiangsu Laboratory of Advanced Functional Materials, College of Material Engineering, Changshu Institute of Technology, Changshu, China; ^3^ State Key Laboratory of Materials-Oriented Chemical Engineering, College of Chemical Engineering, Nanjing Tech University, Nanjing, China

**Keywords:** tetrazole, Gd(III)–Cu(II), crystal structure, chemodynamic therapy, nanobooster

In the original article, there was an error in Figure 6D, page 6, as published. The corrected figure appears below.

**FIGURE 6:**
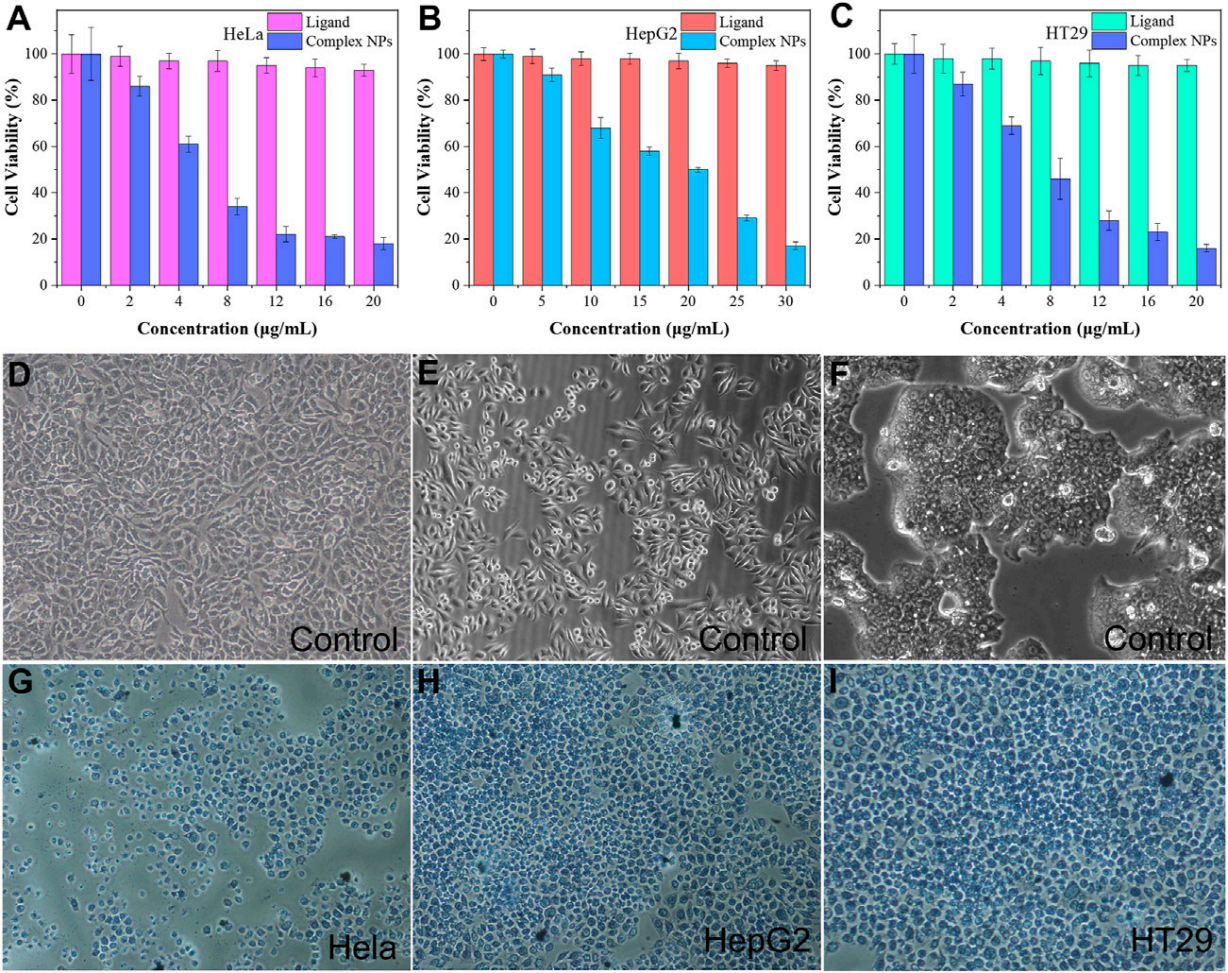
**(A–C)** In vitro MTT assay of HeLa, HepG2, and HT29 cells was treated with ligand H4L and complex NPs; trypan blue fluorescent staining of the control group for **(D)** HeLa cells, **(E)** HepG2 cells, and **(F)** HT29 cells; Complex NPs for **(G)** HeLa cells **(H)** HepG2 cells **(I)** HT29 cells.

In the original article, there was an error in the caption for Figure 6, page 6, as published. The corrected caption appears below.

The authors apologize for these errors and state that this does not change the scientific conclusions of the article in any way. The original article has been updated.

