# Supplementary material for: Rational Design of a Gd(III)–Cu(II) Nanobooster for Chemodynamic Therapy Against Cancer Cells
Source: Front Chem. 2022 Apr 7;10:856495. doi: 10.3389/fchem.2022.856495 (PMC9021535; doi:10.3389/fchem.2022.856495)
Supplement: Supplementary file 1 [file DataSheet1.docx]

**Supporting Information**

**The rational design of a** **Gd(III)-Cu(II) nano-booster for chemodynamic therapy against cancer cells**

Xin-Ya Shi^1^, Ting-Xiao Shen^2^, Ao-Lin Zhang^2^, Li-Tao Tan^2^, Wen-Chang Shen^2^, Hai-Jiang Zhong^2^, Shun-Lin Zhang^3^*, Yu-Lan Gu^1^*, Lei Shen^2^*

*1 Department of oncology, Changshu No.2 People’s Hospital, Changshu, 215500, P.R.China*

*2 Jiangsu Laboratory of Advanced Functional Materials, college of Material Engineering, Changshu Institute of Technology, Changshu, 215500, P.R.China*

*3College of Chemical Engineering, State Key Laboratory of Materials-Oriented Chemical Engineering, Nanjing Tech University, Nanjing 211816, P.R. China.*

*Corresponding authors:

E-mail addresses: guyulan@263.net (Y.L. Gu), Zhangsl93@njteach.edu.cn (S.L. Zhang), leishen@cslg.edu.cn (L. Shen).

Table S1 Main bond length and bond angle (Å and °) of [Gd_2_Cu(L)_2_(H_2_O)_10_]·6H_2_O

| Gd1-O1 | 2.591 (2) | Gd1-O2 | 2.379 (2) |
| --- | --- | --- | --- |
| Gd1-O5 | 2.418 (2) | Gd1-O6 | 2.408 (2) |
| Gd1-O7 | 2.415 (2) | Gd1-O8 | 2.387 (2) |
| Gd1-O9 | 2.518 (3) | Gd1-O3A | 2.430 (2) |
| Gd1-O4A | 2.541 (17) | Cu1-N1 | 1.994 (2) |
| Cu1-N5 | 1.972 (2) | Cu1-N1B | 1.994(2) |
| Cu1-N5B | 1.972 (2) |  |  |
| O2-Gd1-O1 | 52.01 (6) | O2-Gd1-O5 | 73.63 (8) |
| O2-Gd1-O6 | 93.25 (8) | O2-Gd1-O7 | 140.94 (7) |
| O2-Gd1-O8 | 134.45 (6) | O2-Gd1-O9 | 68.47 (7) |
| O2-Gd1-O3A | 86.73 (6) | O2-Gd1-O4A | 129.17 (6) |
| O3A-Gd1-O1 | 133.01 (8) | O3A-Gd1-O9 | 74.86 (7) |
| O3A-Gd1-O4A | 52.31 (6) | O4A-Gd1-O1 | 135.99 (6) |
| O5-Gd1-O1 | 70.74 (7) | O9-Gd1-O1 | 103.63 (7) |
| O4A -Gd1-O1 | 135.99 (6) | O8-Gd1-O4A | 74.53 (6) |
| O6-Gd1-O4A | 137.45(7) | O7-Gd1-O4A | 68.70 (6) |
| O6-Gd1-O1 | 69.63 (7) | O7-Gd1-O1 | 144.23(7) |
| O3A-Gd1-O1 | 133.12 (8) | O4A -Gd1-O1 | 135.99 (9) |
| N5-Cu1-N5B | 180.00(1) | N5-Cu1-N1 | 85.91(8) |
| N5–Cu1-N1B | 94.09(8) | N5-Cu1-N5B | 180.00 (10) |
| N5B–Cu1–N1 | 94.08(8) | N5B-Cu1-N1B | 85.91(8) |

Symmetry code: A: 1+[x, y, z](file:///C:\Users\YANGJIE\AppData\Roaming\Microsoft\Word\symopsfn%20_code_1_545); B: 3[−x](file:///C:\Users\YANGJIE\AppData\Roaming\Microsoft\Word\symopsfn%20_code_2_567), 3-y, 2-z; C: -1+x, [y, z](file:///C:\Users\YANGJIE\AppData\Roaming\Microsoft\Word\symopsfn%20_code_1_455).

Table S2 Hydrogen key parameters (Å and °) for [Gd_2_Cu(L)_2_(H_2_O)_10_]·6H_2_O

| *D-H···A* | *D-H* | *H···A* | *[D···A]* | ∠D-H···A |
| --- | --- | --- | --- | --- |
| O(5)–H(5A) ···O(11) ^#1^ | 0.85 | 2.16 | 2.859(3) | 140 |
| O(5)–H(5B) ···O(12) | 0.85 | 2.18 | 2.670(3) | 116 |
| O(6) –H(6B) ··O(3) ^#2^ | 0.85 | 2.18 | 2.717(3) | 121 |
| O(7) –H(7B) ··O(2) ^#3^ | 0.85 | 2.14 | 2.708(3) | 124 |
| O(9) –H(9B) ··O(10) | 0.85 | 2.52 | 2.909(3) | 109 |
| O(9) –H(9C) ··O(3) ^#4^ | 0.85 | 2.60 | 3.291(3) | 139 |
| O(10)–H(10A) ··N(4) ^#4^ | 0.85 | 2.32 | 2.876(3) | 124 |
| O(10) –-H(10B) ··N(7) ^#5^ | 0.85 | 2.31 | 2.928(3) | 131 |
| O(11) –H(11A) ··O(10) ^#6^ | 0.85 | 2.46 | 2.815(3) | 106 |
| O(12) –H(12A) ··N(3) ^#6^ | 0.85 | 2.60 | 2.917(3) | 103 |

Symmetry code: #1: 3-x, 3-y, 2-z; #2: 5/2-x, 1/2+y, 3/2-z; #3: 7/2-x, 1/2+y, 3/2-z; #4: 5/2-x, 1/2+y, 3/2-z; #5: 5/2-x,-1/2+y,3/2-z; #6:3-x,2-y,2-z

**Fig. S1** XRD patterns of the complex retained in solution for one day (for three days in water).

Fig. S2 DLS of NPs of the complex in aqueous solution retained in 24 h.
